# Saline bolus-based electrical impedance tomography method for rapid bedside assessment of regional lung perfusion during ECMO therapy

**DOI:** 10.1186/s13054-022-04142-6

**Published:** 2022-09-05

**Authors:** Huaiwu He, Jing Jiang, Mengru Xu, Siyi Yuan, Yun Long, Yi Chi, Inéz Frerichs, Zhanqi Zhao

**Affiliations:** 1 Department of Critical Care Medicine, Peking Union Medical College Hospital, Chinese Academy of Medical Sciences, 1 Shuaifuyuan, Dongcheng District, Beijing, China; 2 Department of Critical Care Medicine, Chongqing General Hospital, No.118, Xingguang Avenue, Liangjiang New Area, Chongqing, 401147 China; 3 Department of Anesthesiology and Intensive Care Medicine, University Medical Center of Schleswig-Holstein, Campus Kiel, Kiel, Germany; 4 Department of Biomedical Engineering, Fourth Military Medical University, Xi’an, China; 5 Institute of Technical Medicine, Furtwangen University, Villingen-Schwenningen, Germany

**Keywords:** Lung perfusion, EIT, Electrical bioimpedance, Ventilation–perfusion matching, Extracorporeal membrane oxygenation

Lung perfusion can be assessed using electrical impedance tomography (EIT) and saline bolus injection through central venous catheter (CVC) in the jugular or the subclavian vein in patients [1]. Animal studies had validated this method against other imaging modalities [2]. It was unclear whether lung perfusion could be assessed in patients under extracorporeal membrane oxygenation (ECMO) therapy. On the one hand, a high re-circulation fraction of veno-venous (VV) ECMO could cause a decrease in bolus saline arriving to the lung. On the other hand, the ECMO flow could directly transfer the injected saline from vena cava to aorta. Both the re-circulation fraction of VV ECMO and the flow of veno-arterial (VA) ECMO could impact the dose of saline bolus across the lung. In this letter, we present the use of EIT and saline bolus injection to assess lung ventilation–perfusion in two patients under VV and VA ECMO therapies, respectively.

## Case 1

**Fig. 1:**
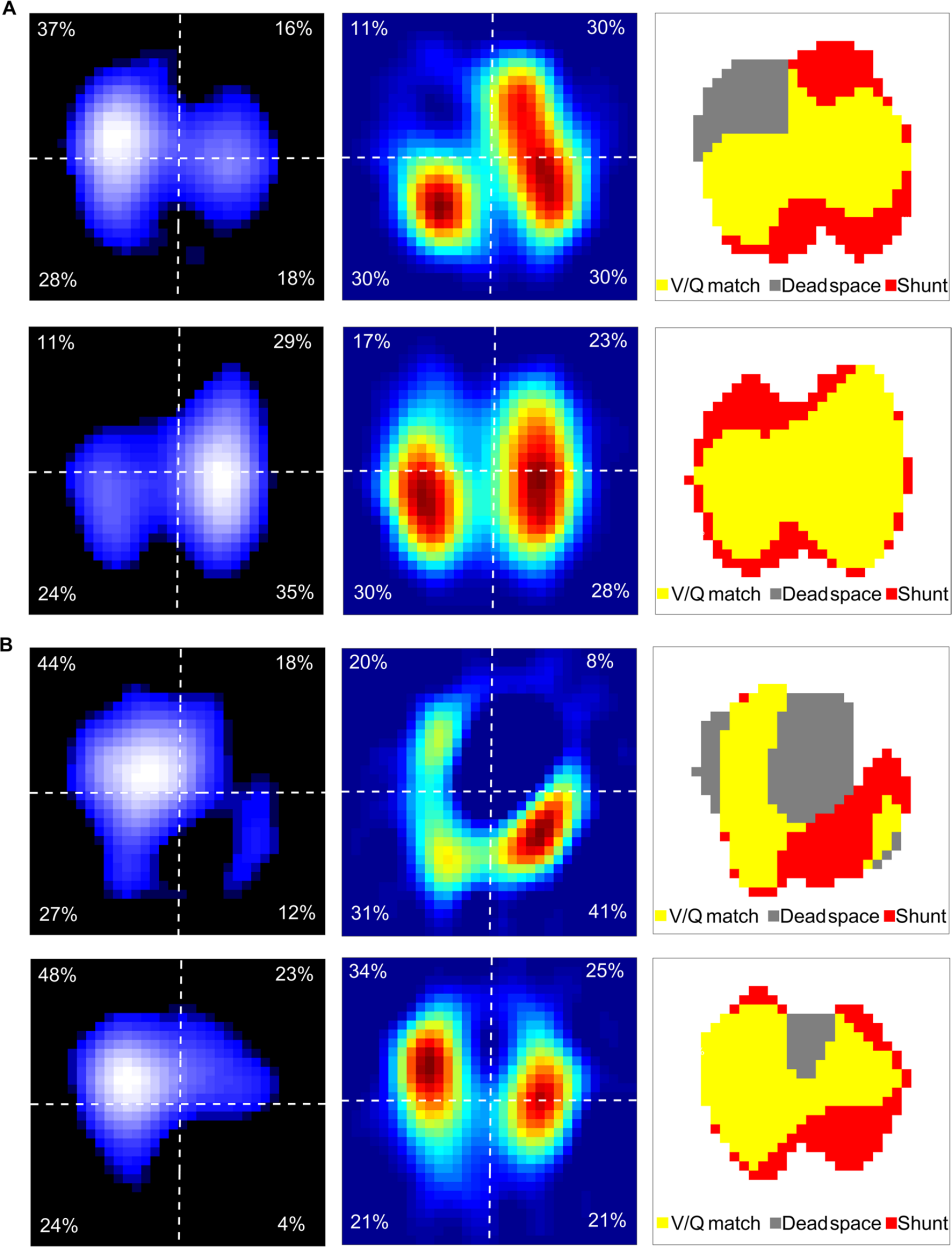
**A** Effect of prone position on ventilation, perfusion and ventilation–perfusion (V/Q) matching in a patient under VV ECMO therapy. First row, supine position. Second row, prone position. First column, functional EIT image showing tidal ventilation distribution. Highly ventilated regions are marked in light blue to white. Distribution percentages are listed in the corresponding regions of interest (quadrants). Second column, functional EIT image showing perfusion distribution. Highly perfused regions are marked in red. Third column, functional EIT image showing the distribution of regional ventilation–perfusion matching. Ventilated regions were defined as pixels with impedance changes higher than 20% of the maximum tidal impedance variation in the functional ventilation image. Perfused regions were defined as pixels higher than 20% of the maximum bolus-related impedance change in the functional perfusion image. Regions with high ventilation and low perfusion are marked in gray (denoted as dead space), low ventilation and high-perfusion regions in red (denoted as shunt), and good ventilation–perfusion matching in yellow (denoted as V/Q match). From supine to prone position, dead space decreased from 17.1 to 0.0%, shunt changed from 28.6 to 25.1%, and V/Q match increased from 54.3 to 74.9%. **B** Effect of thrombolysis on regional perfusion and V/Q in V-A ECMO therapy. Third row, before thrombolysis. Fourth row, after thrombolysis. After thrombolysis, dead space decreased from 36.4 to 8.5%, shunt changed from 27.4 to 28.7%, and V/Q match increased from 36.2 to 62.8%

A 33-year-old woman with pulmonary lymphangiomyomatosis, who had an acute hemoptysis and severe respiratory failure, received mechanical ventilation and VV ECMO treatment. She underwent prone positioning for sputum drainage and improvement in dorsal ventilation. Lung ventilation and perfusion in the supine and prone positions were evaluated using EIT (PulmoVista 500, Dräger Medical, Lübeck, Germany) and saline bolus injection as described previously. Dorsal ventilation increased, and ventral ventilation decreased in the prone position along with a right-to-left redistribution. The ventrodorsal perfusion changes were opposite to ventilation but far less pronounced. Overall, the prone position led to a decrease in dead space in the ventral and shunt in the dorsal regions. This resulted in a better ventilation and perfusion matching (Fig. 1A). Interestingly, the proning also decreased lung perfusion heterogeneity but not the heterogeneity in ventilation distribution (Fig. 1A), which was a behavior different from COVID-19 patients [3]. The patient was successfully weaned from the ventilator and ECMO and transferred to regular ward.

## Case 2

A 62-year-old woman with fatal pulmonary embolism, who received extracorporeal cardiopulmonary resuscitation treatment. Lung perfusion was assessed by saline bolus-based EIT method at the bedside at the time of thrombolysis therapy under VA ECMO. The regional perfusion and ventilation–perfusion matching improved after thrombolysis therapy (Fig. 1B), which was consistent with the improvement in clinical conditions (reduction in right heart size and recovery of circulation).

To our best knowledge, this is the first clinical report using EIT to dynamically assess lung regional perfusion and ventilation–perfusion matching in the ECMO-treated acute respiratory failure or circulatory shock. Conventional radiological lung perfusion assessment by CT pulmonary angiography and dual-energy CT requires patient transport which not only increases the risk but is also difficult under the ECMO treatment. EIT has the potential to estimate both regional lung ventilation and perfusion at the bedside. In the present two cases, the impedance–time curves induced by 10 ml 10% NaCl bolus exhibited a clear continuous impedance decrease limb and trough and were similar to the curves recorded previously without ECMO therapy. The comparison of perfusion images with and without ECMO conducted on the same patients would have been ideal, but it is not available. The maximum drop in VV ECMO was ~ 80% of tidal variation, which was similar to previous patients without ECMO. The maximum drop of impedance in VA ECMO was ~ 45% of tidal variation before breath holding, which indicated a possible loss of saline due to draining from central venous to artery in VA-ECMO. Mendes et al. interrupted blood flood during VV-ECMO therapy before the 10 ml 20% NaCl bolus injection [4]. The bolus was injected in approximately 2–4 s in the right atrium through the pulmonary artery catheter. In contrast with that relatively slow saline injection, we completed our bolus injection in ~ 1 s. This reduced the potential saline lost. Nevertheless, the detailed knowledge and mechanisms are yet to be explored. Further study is required to identify the impact of ECMO on perfusion images via interruption or various blood flood rates. The re-circulation fraction of VV ECMO and effect of saline draining by the VA ECMO should be further determined by establishing the correlation between maximum slope of impedance decrease and blood flow rate. The compute and incorporate the anatomical dead space and cardiac output could provide more accurate EIT map of regional ventilation–perfusion matching [5], which were not considered in the current study.

Overall, the lung regional ventilation and perfusion findings determined by EIT were consistent with the recovery of clinical condition before and after the related treatments. This implies that EIT might have the potential to assess regional perfusion and ventilation–perfusion matching in the ECMO therapy condition. Further study is required to validate the relevance of EIT for diagnosis and clinical decision guidance during ECMO.

## Data Availability

Not applicable.

## Abbreviations

EIT: Electrical impedance tomography
PE: Pulmonary embolism
CTPA: Computed tomography pulmonary angiography
ECMO: Extracorporeal membrane oxygenation
